# Cardio-green butyl acetate targets ergosterol to kill *Candidaalbicans*

**DOI:** 10.1016/j.isci.2026.116886

**Published:** 2026-07-24

**Authors:** Piyush Baindara, Suresh K. Mondal, Dinata Roy, Sourav Chakraborty, Dheeraj Kumar Sarkar, Santi M. Mandal, Gourisankar Ghosh

**Affiliations:** 1 Division of Animal Sciences, University of Missouri, Columbia, MO 65211, USA; 2 National Swine Testing Center, University of Missouri, Columbia, MO 65211, USA; 3 Department of Bioscience and Biotechnology, Indian Institute of Technology Kharagpur, Kharagpur 721302, WB, India; 4 National Institute of Homoeopathy, Block-GE, Sector-III, Salt Lake, Kolkata 700106, India; 5 Tata Institute of Fundamental Research, Hyderabad 500046, Telangana, India; 6 Department of Biochemistry and Molecular Biophysics, University of California, San Diego, 9500 Gilman Dr, La Jolla, CA 92093, USA

**Keywords:** *Candida albicans*, cardio-green butyl acetate, ergosterol, drug-resistance, membrane simulation

## Abstract

The rising incidence of invasive *Candida* infections highlights the urgent need for distinct antifungal agents with improved safety and efficacy. Although several antifungal drugs are clinically available, their associated toxicity and the increasing emergence of drug-resistant *Candida* species often result in poor therapeutic outcomes. In this study, we report the antifungal potential of cardio-green butyl acetate (CaGA), a previously unknown synthesized compound, and investigate its activity and mechanism of action. CaGA exhibited strong fungicidal activity against *Candida albicans*, significantly reducing planktonic cell viability and biofilm biomass. Membrane disruption was demonstrated by protein leakage assays, Nile red and live/dead staining, and scanning electron microscopy. Isothermal titration calorimetry (ITC) confirmed direct interaction between CaGA and ergosterol, the major sterol component of the *C. albicans* membrane. Coarse-grained membrane simulations further revealed preferential interaction with ergosterol-rich domains and pore-like structure formation. Together, these findings identify CaGA as a promising next-generation antifungal candidate with a distinct membrane-targeting mechanism, offering therapeutic potential against drug-resistant *Candida* infections.

## Highlights

• Identifies CaGA as an antifungal candidate
• CaGA kills *C. albicans* planktonic cells and inhibits biofilm formation
• CaGA selectively binds ergosterol, causing pore formation and membrane disruption
• CaGA, a polymethine compound, may form a new antifungal class beyond polyenes

## Graphical abstract

**Figure:**
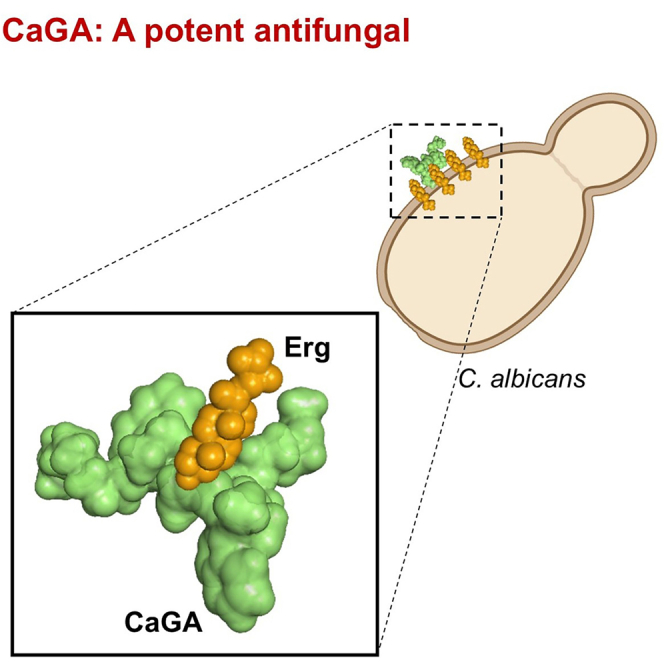

## Teaser

Pharmaceutical compounds formulation; Chemical compound; Biomolecules; Membranes

## Introduction

Fungal infections caused by *Candida* species, particularly *C*. *albicans*, represent a significant and growing global health burden. *C*. *albicans* is responsible for a wide spectrum of diseases ranging from superficial mucosal infections to life-threatening systemic candidiasis, especially in immunocompromised individuals.^1^ The increasing incidence of drug-resistant *Candida* strains, biofilm-associated persistence, and the limited efficacy of existing antifungal drugs have intensified the need for the development of previously uncharacterized, more effective, and safer antifungal agents.^2^ Candida often forms co-infections with bacterial strains, leading to more severe and complicated infections. This synergistic interaction can worsen patient outcomes and complicate treatment strategies.^3^^,^^4^ Among the currently available antifungals, amphotericin B (AmB) remains a gold-standard treatment due to its broad-spectrum activity and fungicidal nature.^5^^,^^6^ AmB exhibits anti-*Candida* activity by binding to ergosterol, a key component of fungal cell membranes, and forming transmembrane pores that lead to ion leakage and cell death.^7^ Notably, clinical uses of AmB are limited due to serious dose-limiting toxicities, particularly nephrotoxicity and infusion-related side effects.^8^^,^^9^ Overall, the emergence of rapid drug resistance, along with the lack of available drugs with safety profiles, highlights the urgent need for alternative compounds to combat pathogenic *C*. *albicans*. Cardio-green (C_43_H_47_N_2_NaO_6_S_2_) (CaG), also known as indocyanine green, is an FDA (Food and Drug Administration)-approved tricarbocyanine dye widely utilized as a near-infrared fluorescent probe in clinical diagnostics and imaging. It has been extensively applied in cardiac output monitoring, hepatic function assessment, ophthalmic angiography, and intraoperative imaging due to its favorable safety profile, strong plasma protein binding, and deep tissue penetration capabilities.^10^^,^^11^ Interestingly, CaG is reported to bind specifically to lipoprotein and lipids, including sterols such as cholesterol.^12^ In the present study, we have synthesized a modified derivative of CaG, cardio-green butyl acetate (CaGA), to enhance its stability, lipophilicity, membrane binding, and biological activity while investigating the anti-*Candida* properties in comparison to AmB. We employed an integrative approach combining *in vitro* assays and coarse-grain molecular dynamics (MD) simulations using asymmetric ergosterol-rich bilayers mimicking the *C. albicans* membrane to characterize CaGA’s mechanism of action. By presenting detailed experimental and simulation-based evidence, this study suggested that CaGA might be a foundation for next-generation antifungal classes with the potential to overcome the limitations of current therapies.

## Results

**Figure 1:**
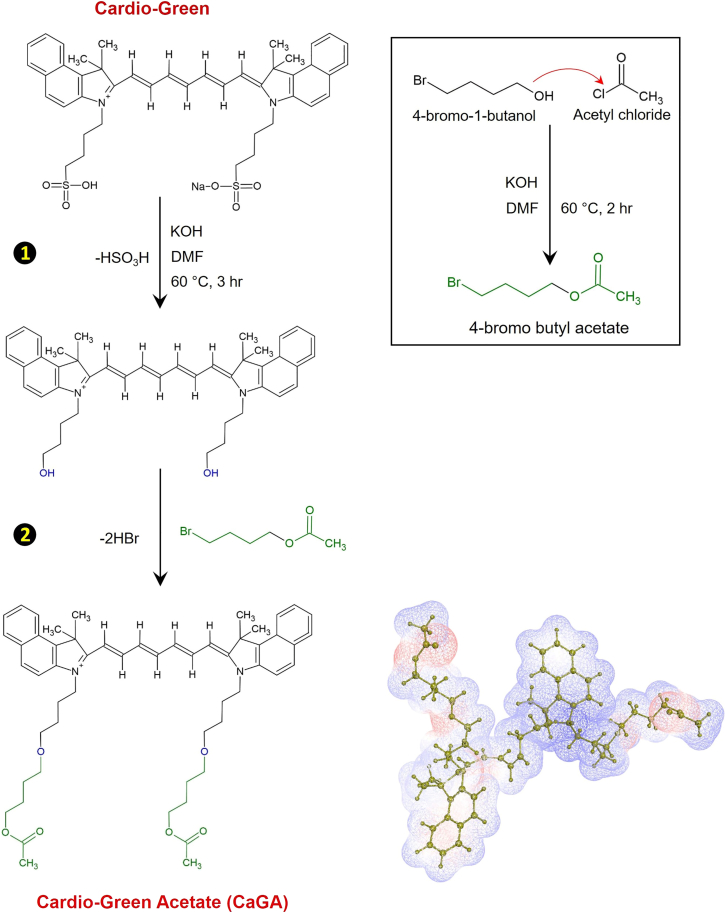
Synthesis and structural confirmation of CaGA (A) Two-step synthetic route showing conversion of CaG into its butyl acetate-functionalized derivative (CaGA). Step 1: formation of bromo-butyl acetate intermediate; Step 2: substitution reaction yielding CaGA.

The final product (Figure 1) was purified and confirmed using ^1^H nuclear magnetic resonance (^1^H NMR) spectroscopy, which validated the successful modification of CaG into its butyl acetate-functionalized derivative CaGA. ^1^HNMR: 1.62–1.69, 6H (m), 1.84–1.86, 3H (m), 2.07, 6H (S), 2.08, 4H (S), 2.46–2.56, 8H (m), 2.75, 2H (S), 2.89, 2H (S), 3.29, 16H (S), 3.51–3.54, 4H (t), 4.02, 2H (S), 5.02–5.06, 1H (m), 5.26–5.42, 1H (m), 6.02–6.06, 2H (m), 6.42–6.49, 2H (m), 7.74–7.86, 5H (m), 7.93–8.21,5H (m) (Figure S1).

### CaGA efficiently kills *C*. *albicans*

**Figure 2:**
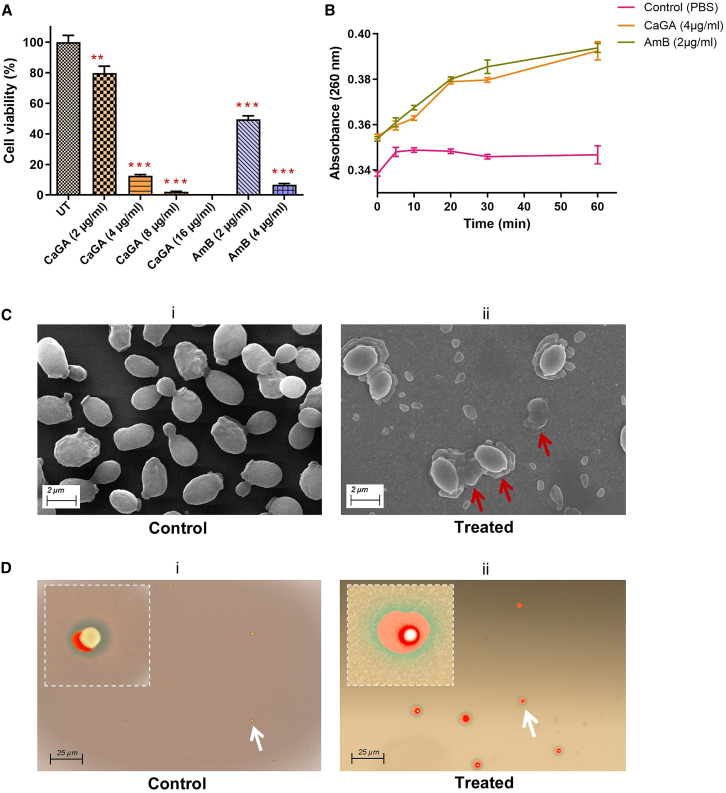
Antifungal activity of CaGA against *C. albicans* (A) CFU assay reveals a dose-dependent inhibition of *C. albicans*, where complete inhibition was observed at 16 μg/mL of CaGA. (B) Intracellular leakage of *C. albicans* with 4 μg/mL of CaGA for 0, 5, 10, 20, 30, and 60 min. AmB was used as a positive control. (C) SEM micrograph of untreated and treated *C. albicans* with 4 μg/mL of CaGA. (D) Nile red staining assay of *C. albicans* at 4 μg/mL of CaGA. Untreated (PBS) *C. albicans* was used as a negative control. All the experiments were performed three times independently in triplicate, while statistical significance was considered at the level of *p* < 0.05. Error bars represent standard deviation (SD).

The antifungal efficacy of CaGA against *C*. *albicans* was examined by treating planktonic cells, followed by plating on agar media and colony counting to determine the minimum inhibitory concentration. Treatment with CaGA led to a significant, dose-dependent reduction in colony-forming units (CFUs), indicating its inhibitory effect on proliferation increases with concentration. Quantitative analysis revealed a significant reduction of more than 85% and 99% of viable colonies at 4 μg/mL and 8 μg/mL of CaGA, respectively, when compared to untreated controls (Figure 2A). These results demonstrate that CaGA efficiently kills *C*. *albicans* and establishes its potential as an antifungal agent, comparable to the frontline antifungal drug, AmB.

### CaGA targets membrane-associated killing mechanisms

To further confirm the anti-*Candida* activity of CaGA and to assess the effect on *C*. *albicans* membrane integrity, a protein leakage assay was performed by measuring UV absorbance at 260 nm. Treatment of *C*. *albicans* with CaGA at 4 μg/mL for 5, 10, 20, 30, and 60 min time points exhibited a significant increase in extracellular protein content when compared to untreated controls (Figure 2B). The elevated absorbance at 260 nm indicates leakage of intracellular proteins or protein-nucleic acid complexes into the extracellular environment, suggesting compromised cell membrane integrity. This leakage correlated with a significant reduction in cell viability, confirming that CaGA exerts its anti-*Candida* effect by disrupting the cell membrane, leading to cell death.

Further, scanning electron microscopy (SEM) was performed to examine the morphological impact of the CaGA on *C*. *albicans* cells. Notably, *C*. *albicans* cells treated with CaGA at 4 μg/mL exhibit significant signs of structural damage in comparison to the control cells. SEM images revealed surface deformities such as wrinkling, membrane blebbing, cell shrinkage, and irregular cell shapes, along with disrupted cell membranes, indicative of compromised cellular integrity (Figure 2C).

Additionally, Nile red staining was performed on treated *C*. *albicans* cells to check if membrane sterols or intracellular lipids are altered or disrupted upon CaGA treatment and contribute to the killing mechanism. Interestingly, the increased Nile red fluorescence observed in *C*. *albicans* cells treated with 4 μg/mL of CaGA suggests the disruption of membrane lipid domains, likely due to altered ergosterol content or membrane fluidity, consistent with a membrane-targeted antifungal mechanism (Figure 2D). Additionally, fluorescence microscopy revealed altered membrane morphology and patchy distribution of lipid domains in treated cells compared to untreated controls. These observations support the hypothesis that CaGA might interact with ergosterol in the *C*. *albicans* membrane, potentially perturbing membrane integrity and contributing to its antifungal activity. Together, these findings demonstrate that CaGA exerts potent antifungal effects against *C*. *albicans* by disrupting membrane integrity, inducing morphological damage, and thus reducing viability.

### CaGA efficiently eradicates *C. albicans* biofilm

**Figure 3:**
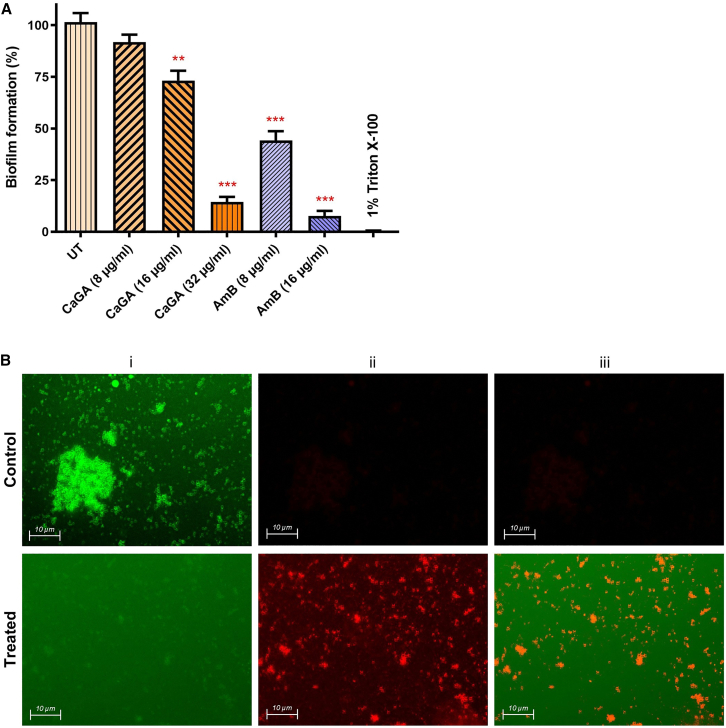
Biofilm eradication potential of CaGA (A) Crystal violet assay shows efficient eradication of *C. albicans* biofilms in comparison to the AmB. *C. albicans* treated with PBS 1% Triton X-100 were used as negative and positive controls, respectively. (B) Fluorescence microscopic image of live/dead biofilm eradication assay of untreated biofilms (upper panel), and treated biofilms (lower panel) for 1 h at 4 μg/mL of CaGA (i) sytox green, (ii) propidium iodide, and (iii) merged. Error bars represent SD, while statistical significance is considered at the level of *p* < 0.05. All the experiments were performed three times independently in triplicate.

The ability of CaGA to eradicate *C*. *albicans* biofilm was evaluated using the crystal violet (CV) assay. Treatment with CaGA at a 32 μg/mL of CaGA concentration resulted in a significant reduction in biofilm biomass of *C*. *albicans* compared to untreated controls (Figure 3A). Interestingly, the biofilm removal efficacy of CaGA was comparable to that of AmB, which was used as a positive control. Quantitative analysis showed that *C*. *albicans* biofilm biomass was reduced by approximately 25% and 85%, respectively, at 16 μg/mL and 32 μg/mL concentration of CaGA. At the same time, AmB exhibits about 50% and 90% reduction in *C*. *albicans* biofilm mass at 8 μg/mL and 16 μg/mL of concentration, respectively (Figure 3A). These results confirmed the potent antibiofilm activity of CaGA against *C*. *albicans* biofilm. Further, the viability of CaGA-treated *C*. *albicans* biofilms was evaluated using the live/dead BacLight staining assay. Untreated control biofilms exhibited predominantly green fluorescence (SYTO9), indicating intact cell membranes and high viability (Figure 3B). In contrast, *C. albicans* biofilms treated with CaGA at a 4 μg/mL concentration for 60 min revealed a significant increase in red fluorescence (PI), indicative of compromised cell membrane and cell death (Figure 3B). Overall, these results demonstrate that CaGA exerts potent antifungal effects on *C*. *albicans* biofilms, compromising membrane integrity and thus significantly reducing cell viability.

### CaGA binds to ergosterol, leading to *C. albicans* cell death

**Figure 4:**
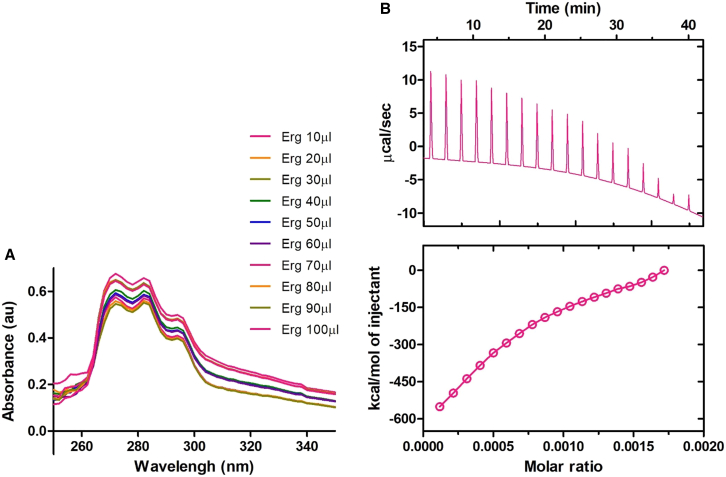
Biophysical characterization of CaGA-ergosterol interaction (A) UV-visible absorption spectra of CaGA in the presence of increasing ergosterol concentrations showing a decrease and red shift of the ergosterol peak at 282 nm, consistent with complex formation. (B) ITC profile shows an exothermic binding event (ΔH = −3.02 × 10^3^ cal/mol·K, K = 3.2 × 10^4^ M^−1^). The thermodynamic parameters indicate a spontaneous, enthalpy-driven interaction involving hydrophobic and hydrogen-bonding forces between CaGA and ergosterol.

The interaction between *C*. *albicans* cell membrane sterol, ergosterol, and CaGA was primarily investigated using UV absorbance spectroscopy. Upon incubation of different concentrations of ergosterol with CaGA at a 100 μg/mL concentration, significant changes in the UV absorbance spectrum were observed compared to ergosterol alone. Specifically, a notable shift and/or decrease in the characteristic ergosterol absorbance peak at 282 nm indicated binding or complex formation between CaGA and ergosterol (Figure 4A). These spectral changes provide strong evidence of a direct interaction of CaGA with ergosterol, which may underlie its mechanism of disrupting *C*. *albicans* cell membranes and thus exerting antifungal effects.

Isothermal titration calorimetry (ITC) was performed to further confirm the interaction between CaGA and ergosterol. Key parameters for interaction, including Gibbs free energy (ΔG), enthalpy change (ΔH), entropy change (TΔS), binding affinity (K), and stoichiometry (n), were quantitatively assessed using ITC in the present study. The binding isotherms and thermograms demonstrated a strong interaction between the CaGA and ergosterol, with an estimated binding constant (*K*) of 3.20 × 10^4^ M^−1^. Notably, the binding event was characterized as an exothermic reaction, and the enthalpy of binding was −3.02 × 10^3^ cal/mol·K (Figure 4B). The significant heat changes recorded throughout the titration reflect the distinct thermodynamic signature of the interaction, revealing strong, specific non-covalent binding between the CaGA and ergosterol. Collectively, these findings confirm the high binding affinity and reveal the underlying thermodynamic forces governing the interaction, highlighting the potential of CaGA as a potent binder to ergosterol, possibly contributing to the antifungal mechanism of CaGA against *C*. *albicans*.

### Translocation of CaGA across the asymmetric *C*. *albicans* membrane

**Figure 5:**
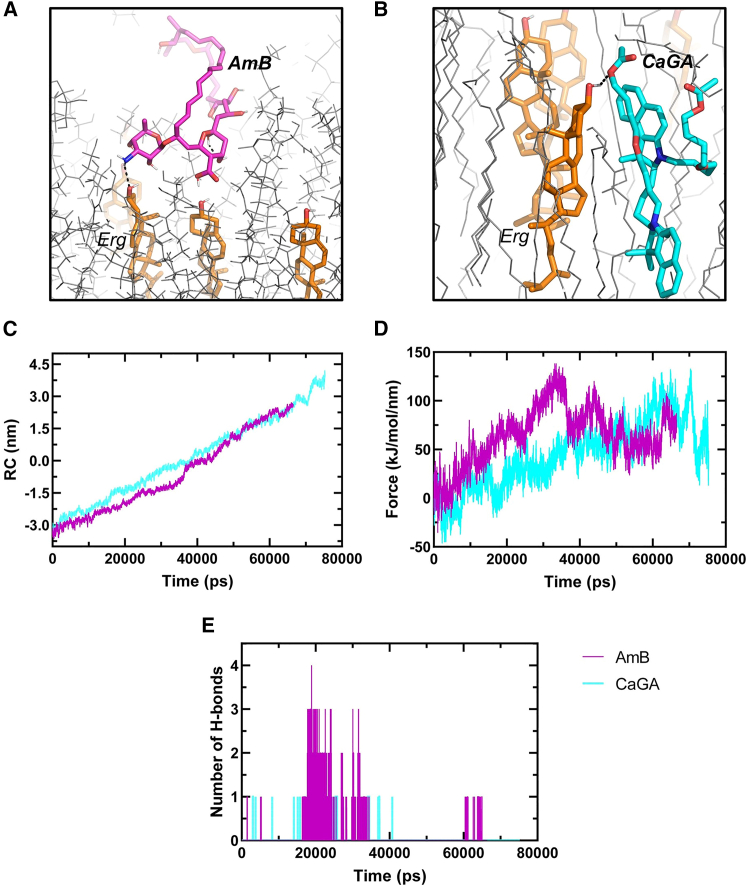
Comparative MD simulation of AmB and CaGA interactions with ergosterol-rich membranes (A) Snapshot shows AmB (magenta) inserting deeply into the ergosterol-rich bilayer (ergosterol in orange), forming a transmembrane channel-like arrangement. (B) Snapshot shows CaGA (cyan) remaining near the membrane surface while engaging ergosterol via hydrogen bonding and hydrophobic contacts, indicating a surface-associated, non-pore-forming mode of action. (C) RC profiles from steered MD simulations illustrate the relative depth of membrane penetration over time for AmB and CaGA. (D) Corresponding force profiles show that AmB requires a higher pulling force to cross the bilayer, reflecting stronger lipid disruption, whereas CaGA maintains lower energy perturbations consistent with membrane thinning rather than pore formation. (E) Time evolution of hydrogen bonds, demonstrating transient but frequent interactions for CaGA compared with AmB.

**Figure 6:**
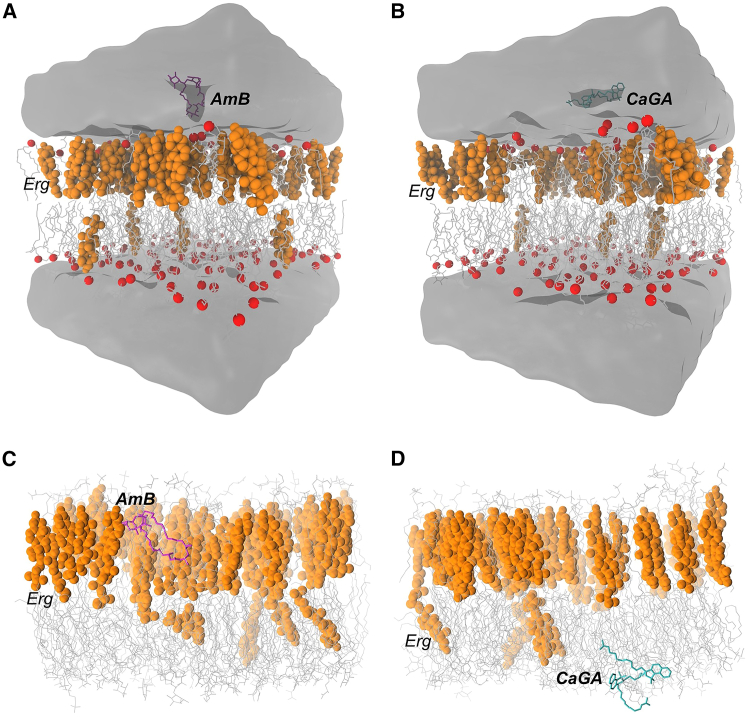
Representative membranes showing CaGA and AmB embedded in the *C. albicans* bilayer (A) Molecular system of AmB is inserted above the outer membrane (OT) region. (B) Molecular system of CaGA near the OT region. The ergosterols are shown in orange spheres, and other lipids are indicated as lines. The phosphate head is shown in red spheres, and the water molecules are represented as transparent surfaces in gray color. For SMD, the compounds were steered from the outer membrane (OT) to the inner membrane (IN) region. The starting (C) and final (D) conformations of CaGA while translocating the cell membrane of *C. albicans*.

To investigate the membrane translocation behavior and thermodynamic feasibility of CaGA, steered molecular dynamics (SMD) simulations were conducted while AmB was used as a comparative control. To generate a force versus distance curve profile, the center of mass of the CaGA and AmB was pulled along the z axis, spanning from −4 nm to +4 nm, which defined the reaction coordinate (RC) across the fungal bilayer (Figure 5). A constant pulling velocity of 0.1 nm/ns and a harmonic force constant of 100 kJ mol^−1^nm^−2^ were applied to simulate transport from the outer membrane surface to the inner leaflet region. The force-distance profiles derived from SMD trajectories revealed distinct energy barriers for both CaGA and AmB (Figure 5). Notably, CaGA exhibited its highest force peak during egress from the membrane interior, suggesting a strong retention within the bilayer and a potentially deeper membrane interaction. Specifically, a peak force of 133 kJ mol^−1^ nm^−1^ was required to pull CaGA out of the membrane, indicating significant bilayer engagement (Videos S1 and S2). In contrast, AmB demonstrated its maximum force barrier of 138 kJ mol^−1^ nm^−1^ during entry, suggesting it faces a higher energy requirement upon initial membrane insertion (Figure 6). These findings underscore the distinct membrane-interaction dynamics of CaGA, highlighting its potential for stable bilayer insertion and membrane-disruptive antifungal activity against *C*. *albicans*.

Video S1. Coarse-grained membrane simulation of AmB binding ergosterol and translocating across the *C. albicans* membrane

Video S2. Coarse-grained membrane simulation of CaGA binding ergosterol and translocating across the *C. albicans* membrane

### CaGA kills *C. albicans* by a distinct interaction with ergosterol

**Figure 7:**
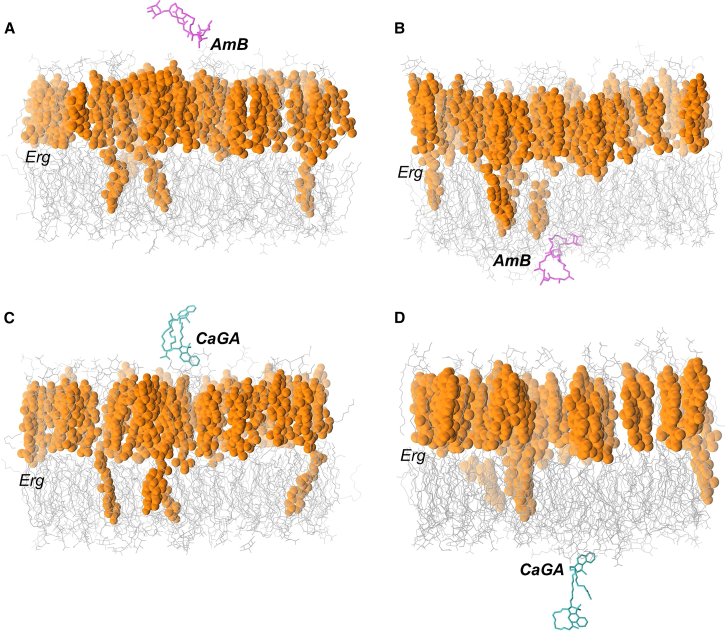
Initial and final conformations of AmB and CaGA in *C. albicans* during SMD (A) The starting and (B) final conformations of AmB while translocating the *C. albicans* membrane. (C) The starting and (D) final conformations of CaGA while translocating the *C. albicans* membrane.

To elucidate the molecular basis of antifungal action, all-atom MD simulations were conducted to investigate the interaction of CaGA with ergosterol-containing asymmetric membranes representative of *C. albicans*. Interestingly, CaGA is primarily localized at the membrane interface, engaging in extensive hydrogen bonding and hydrophobic interactions with ergosterol’s polar head groups and sterol rings. CaGA disrupted the lateral organization of ergosterol and surrounding phospholipids, leading to membrane destabilization and altered fluidity. This disruption potentially compromises membrane integrity, thus exhibiting killing. On the other hand, AmB exhibited strong affinity for ergosterol-rich membrane regions, inserting deeply into the lipid bilayer. This interaction facilitated the formation of stable transmembrane channels, characterized by the aggregation of AmB molecules surrounding ergosterol clusters, consistent with the classical pore-forming mechanism. Moreover, both compounds exhibited the highest degree of ergosterol engagement during the 20–35 ns window of the SMD trajectory, suggesting that this time frame corresponds to a critical interaction phase as the molecules traverse the hydrophobic core of the membrane (Figure 7). Notably, CaGA formed hydrogen bonds with ergosterol, primarily mediated via its oxygen atoms, indicating specific and directional interactions stabilizing its position within the lipid bilayer (Figure 5). Overall, the membrane simulations revealed that CaGA binding induces significant local membrane thinning and increased lipid disorder via ergosterol, which is also possibly involved in the promotion of ion permeability and cell leakage. Together, these simulation results provide atomistic insights into how CaGA differentially targets *C*. *albicans* through ergosterol binding, advancing understanding of its antifungal mechanism of action and informing rational design of emerging therapeutics.

## Discussion

The growing prevalence of drug-resistant *Candida* infections continues to challenge current antifungal therapy. AmB, a polyene macrolide, remains a benchmark treatment because of its broad-spectrum activity and potent fungicidal action. However, its severe nephrotoxicity and infusion-related adverse effects limit clinical use, emphasizing the need for safer antifungal agents with comparable efficacy.^5^^,^^6^ In this context, we developed and characterized CaGA, a previously unknown synthesized derivative of indocyanine green, and evaluated its antifungal mechanism using both experimental assays and MD simulations. Our findings establish that CaGA exerts strong antifungal activity against *C*. *albicans* by directly compromising the fungal membrane. CFU assays and live/dead staining revealed a clear dose-dependent reduction in viability (Figures 2A and 2B), while biofilm assays showed that CaGA significantly reduced biofilm biomass, performing comparably to AmB (Figure 3). This dual effect on planktonic and biofilm cells highlights CaGA’s potential therapeutic advantage, since biofilms are typically more resistant to conventional antifungals.^13^ Mechanistically, multiple lines of evidence indicate that CaGA’s antifungal activity is membrane-centric and ergosterol-dependent. The rise in UV absorbance at 260 nm following CaGA treatment suggests leakage of intracellular components due to membrane rupture (Figure 2B). SEM further confirmed severe morphological distortions, including wrinkling, shrinkage, and cell deformation (Figure 2C). Importantly, Nile red staining demonstrated increased lipid-domain fluorescence and patchy distribution patterns in treated cells (Figure 2D), consistent with altered ergosterol-rich microdomains and membrane fluidity changes. Together, these observations support a model in which CaGA interacts directly with fungal sterols rather than acting as a nonspecific detergent. Direct ergosterol-binding assays further substantiate this conclusion. Next, UV-visible spectroscopy revealed spectral shifts upon ergosterol addition, and ITC demonstrated a strong, exothermic binding reaction (ΔH = −3.02 × 10^3^ cal/mol·K) with a moderate binding affinity (K = 3.2 × 10^4^ M^−1^) (Figures 4A and 4B). This thermodynamic signature suggests that CaGA forms a stable non-covalent complex with ergosterol, driven by hydrophobic and hydrogen-bonding interactions. Additionally, MD simulations provided deeper mechanistic insight into how these interactions influence membrane structure. CaGA localized near the membrane surface, engaging in multiple hydrogen bonds with ergosterol’s hydroxyl groups and disrupting local lipid packing. Unlike AmB, which penetrates the bilayer and forms transmembrane ion channels, CaGA remained surface-associated and induced localized thinning of the membrane. This non-pore-forming but membrane-destabilizing mechanism likely promotes leakage and ion imbalance without creating permanent pores. The lower energetic barrier observed in steered MD simulations implies that CaGA associates with ergosterol-rich regions with minimal perturbation of surrounding lipids, potentially leading to reduced off-target toxicity compared with AmB.^14^^,^^15^ Taken together, these findings define CaGA as a membrane-selective antifungal agent that kills *C. albicans* by destabilizing ergosterol-dependent lipid organization. This is mechanistically distinct from AmB and other polyenes, which form rigid transmembrane channels that can also disrupt mammalian cholesterol membranes.^16^ CaGA’s preferential interaction with ergosterol over cholesterol could therefore provide a broader therapeutic window. The strong antibiofilm activity observed further aligns with its membrane-targeted mechanism. Biofilm formation in *Candida* depends on intact plasma membrane signaling, adhesion proteins, and lipid-domain organization. Disruption of ergosterol-rich microdomains likely impairs these functions, weakening biofilm cohesion and viability. The combined activity against both planktonic and biofilm cells suggests that CaGA can overcome one of the primary challenges in antifungal therapy, biofilm-associated tolerance to antifungal agents.^17^ Although these results position CaGA as a promising antifungal candidate, further investigation is warranted. Future work should evaluate its selectivity index, *in vivo* safety, and pharmacokinetics to confirm therapeutic potential. Structural analogs of CaGA could also be explored to fine-tune ergosterol affinity and optimize membrane selectivity. In summary, CaGA acts as a distinct, ergosterol-targeting antifungal that disrupts fungal membranes through a non-pore-forming, membrane-destabilizing mechanism. This dual action-potent fungicidal activity and biofilm disruption, combined with likely lower cytotoxicity underscores its potential as a next-generation antifungal agent capable of addressing the limitations of existing therapies. Overall, this study identifies CaGA as a distinct antifungal molecule that targets fungal membranes through specific interaction with ergosterol. Its distinct mechanism and membrane-selective action highlight potential advantages in safety and efficacy compared with existing agents. Overall, CaGA shows strong promise as a next-generation therapeutic candidate for *C*. *albicans*, including biofilm-associated and drug-resistant infections.

### Limitations of the current study

This study provides strong experimental and computational evidence that CaGA acts as a distinct ergosterol-targeting antifungal. Still, a few areas warrant further investigation. The work focused on a single *C. albicans* strain to establish proof of mechanism; testing across clinical and non-*albicans* species will help define the broader activity range. Next, ergosterol binding was confirmed by multiple complementary methods; we did not measure total ergosterol content before and after treatment, as this would have been largely redundant. Further, cytotoxicity toward mammalian cells was not evaluated here, but CaGA’s preference for ergosterol over cholesterol suggests a favorable safety profile to be verified in future *in vitro* studies. Likewise, while live/dead and CFU assays showed consistent results, formal statistical correlation was not performed since both already supported the same trend. Finally, *in vivo* studies will be needed to confirm safety, pharmacokinetics, and resistance potential under physiological conditions. Overall, these limitations define clear next steps for advancing CaGA from a validated *in vitro* candidate to a promising antifungal lead.

## Resource availability

### Lead contact

Further information and requests for resources and reagents should be directed to and will be fulfilled by the lead contact, Santi M. Mandal (smmandal@ucsd.edu).

### Materials availability

This study did not generate new or unique reagents.

### Data and code availability

• Data: None.
• Code: None.
• All other items: None.

## Acknowledgments

P.B. and D.R. acknowledge the National Swine Testing Center and the Division of Animal Sciences, University of Missouri, Columbia, United States, for providing the necessary space and facilities for this work.

## Author contributions

S.M.M. conceptualized the work. P.B. wrote the manuscript and prepared the illustrations. S.K.M. synthesized and characterized the compound. S.C. performed the activity assay. D.R. and D.K.S. performed the membrane simulations. S.M.M. and G.G. proofread and supervised the whole work.

## Declaration of interests

The authors declare no competing interests.

## STAR★Methods

### Key resources table

**Table:** 

REAGENT or RESOURCES	SOURCE	IDENTIFIER
**Fungal strain**		

*C. albicans* SJ11	This study	N/A

**Chemicals**		

Sabouraud Dextrose Agar	HiMedia	MH063
Sabouraud Dextrose Broth	HiMedia	MH033
Yeast Extract Peptone Dextrose	HiMedia	M671
Dimethyl formamide	Merk	68-12-2
Potassium hydroxide	Merk	1310-58-3
Acetyl chloride	Merk	75-36-5
4-bromo-1-butanol	Sigma Aldrich	95517
DMSO	Merk	67-68-5
Phosphate buffer saline	Millipore Sigma	P2272-500 ML
RPMI-1640 medium	Millipore Sigma	R1145-500 ML
Glucose solution	Millipore Sigma	492-62-6
Crystal violet	Merk	548-62-9
Methanol	Merk	67-56-1
Glacial acetic acid	Merk	64-19-7
Live/Dead *Bac*Light Bacterial Viability Kit	Invitrogen	L7012
Poly-L-lysine	Sigma Aldrich	A-005-C
Nile Red	Sigma Aldrich	72485-100 MG
Ergosterol	Sigma Aldrich	E6510-5G

**Software**		

CHARMM-GUI webserver	https://www.charmm-gui.org	N/A
GROMACS	www.gromacs.org	N/A
PyMOL	https://www.pymol.org	N/A

### Method details

#### *Candida* strain and media

The target fungal strain, *C*. *albicans* SJ11, was originally isolated from a patient diagnosed with fungal keratitis.^18^ The strain was maintained on Sabouraud Dextrose Agar (SDA) slants at 4 °C and subcultured periodically to ensure viability. The strain was grown on SDA plates for 24–48 h at 30 °C to obtain single colonies. For planktonic culture, a single colony was inoculated into Sabouraud Dextrose Broth (SDB) and incubated at 30°C with shaking at 180 rpm until the mid-logarithmic phase (OD_600_ ≈ 0.6–0.8). SDA, SAB, and YPD were purchased from HiMedia, India.

#### Antibiotics and other chemicals

Chemicals used in the synthesis of CaGA include Dimethyl formamide (DMF), Potassium hydroxide (KOH), Acetyl chloride (CH_3_COCl), and 4-bromo-1-butanol (C_4_H_9_BrO), which were purchased from Merck India Private Limited, India, and Sigma Aldrich, USA.

#### Synthesis of the compounds and structure determination

The synthesis of CaGA was carried out in the following two steps.

Step 1: Synthesis of Bromo Butyl Acetate

Equimolar amounts of acetyl chloride and 4-bromo-1-butanol (1:1 M ratio) were reacted in the presence of 1.5 equivalents of potassium hydroxide (KOH) as a base, using 5 mL of anhydrous dimethylformamide (DMF) as the reaction solvent. The reaction mixture was refluxed at 60 °C for 2 h under constant stirring in a round-bottom flask. The progress of the reaction was monitored by thin-layer chromatography (TLC). Upon completion, the product was purified via silica gel column chromatography, yielding bromobutyl acetate as an intermediate (Figure 1).

Step 2: Synthesis of CaGA

In a separate reaction, CaG was treated with KOH in DMF and heated at 60 °C for 3 h. The reaction facilitates the release of sulphonic acid, converting it into reactive hydroxyl moieties. The resulting alcoholic derivative of CaG was then reacted with the previously synthesized bromobutyl acetate, followed by nucleophilic substitution conditions. The hydroxyl groups on the CaG derivative attack the electrophilic carbon of the bromo ester, releasing hydrobromic acid (HBr), resulting in the final product CaGA (Figure 1).

#### Antifungal assay

To evaluate the antifungal activity of CaGA against *C*. *albicans*, a colony-forming unit (CFU) inhibition assay was performed as described earlier, with some modifications.^19^ Cells were cultured in SDB at 30 °C with shaking at 200 rpm until the mid-logarithmic phase (OD_600_ ∼0.6–0.8). Cells were then harvested by centrifugation at 5000 rpm for 5 min, washed twice with sterile phosphate-buffered saline (PBS), and resuspended to a final concentration of ∼1 × 10^6^ CFU/mL. CaGA was freshly prepared in sterile DMSO and further diluted in PBS to achieve the desired concentrations, ranging from 2 to 32 μg/mL. Aliquots of 100 μL of the fungal suspension were mixed with 100 μL of different CaGA concentrations in a 96-well plate. Frontline antifungal drug, AmB used as a positive control, while PBS containing equivalent DMSO concentrations was used as a negative control. Treated *C*. *albicans* cells were incubated at 30 °C for 2 h. After incubation, treated samples were serially diluted 10-fold in PBS and subsequently plated on SDA plates. Plates were incubated at 30 °C for 24–48 h. Colonies were counted manually, and CFU/mL was calculated. The minimum anti-*Candid*a concentration (MFC) was defined as the lowest concentration of CaGA that resulted in ≥99.9% reduction in viable colony count compared to the untreated control.

#### Protein leakage assay

To assess membrane integrity upon CaGA treatment, a UV absorbance protein and nucleic acid leakage assay was performed as described earlier.^20^ Cells were cultured in SDB at 30 °C with shaking until mid-logarithmic phase (OD_600_ ∼0.6–0.8). Cells were harvested by centrifugation at 5000 rpm for 5 min, washed twice with sterile PBS, and resuspended. Next, cell suspensions were treated with 4 μg/mL CaGA, while PBS alone and AmB at 2 μg/mL were used as negative and positive controls, respectively. After 2 h of incubation at 30 °C, samples were centrifuged at 8000 rpm for 10 min. The supernatants were collected and analyzed for absorbance at 260 nm using a UV-Vis spectrophotometer (Thermo Fisher Scientific, USA). An increase in absorbance was interpreted as indicative of cellular leakage of nucleic acids and associated proteins, reflecting membrane disruption.

#### Crystal violet assay

Biofilm formation by *Candida* was quantified using the crystal violet (CV) assay as described earlier.^20^ Briefly, *Candida* cells were cultured overnight in yeast extract peptone dextrose (YPD) broth at 30°C–37 °C with shaking. The culture was then adjusted to an optical density of 0.6–0.8 (∼10^6^-10^7^ CFU/mL) at 600 nm (OD_600_) in fresh RPMI 1640 medium supplemented with 2% glucose. Aliquots of 100 μL of the standardized cell suspension were then added to sterile, flat-bottom 96-well polystyrene microtiter plates. Cultures were then allowed to grow and form biofilm in static conditions while incubated at 37 °C for 48 h. After incubation, non-adherent cells were carefully removed by gently washing the wells with sterile PBS three times. The adhered biofilms were then fixed with 100 μL of methanol for 15 min. After removal of methanol, plates were air-dried, and biofilms were subsequently stained with 100 μL of 0.1% (w/v) CV solution for 15 min at RT. Next, excess CV was removed by washing the wells with sterile PBS twice, and the plates were then air-dried completely. To quantify biofilm biomass, the bound CV was solubilized by adding 150 μL of 33% (v/v) glacial acetic acid to each well, and the plate was incubated for 15 min with gentle shaking. Finally, the absorbance of the resulting solution was measured at 570 nm using a microplate reader (Thermo Fisher Scientific, USA).

#### Live/dead staining and detection

To evaluate *C*. *albicans* viability within biofilms, the Live/Dead *Bac*Light Bacterial Viability Kit was used (Invitrogen L7012). *C*. *albicans* biofilm was grown on sterile glass coverslips under appropriate culture conditions for 48 h. Following incubation, non-adherent cells were removed by gentle washing of the biofilms three times with PBS. The staining solution of SYTO9 and propidium iodide PI (1:1 volume ratio) was applied to the CaGA-treated and untreated biofilms and incubated for 15 min at RT in the dark, and subsequently rinsed with PBS to reduce background staining. Biofilms were then immediately observed and imaged under a fluorescence microscope (Zeiss Microsystems, USA).

#### Sample preparation for SEM

A mid-exponential phase culture of *C*. *albicans* (approximately 10^6^-10^8^ CFU/mL) was used to prepare the SEM samples. The cells were harvested by centrifugation at 8000 rpm for 10 min, followed by two washes with PBS to remove residual media and debris. The cell pellet was then resuspended in PBS to achieve an approximate concentration of 10^8^ CFU/mL. For treatment, high-density *C*. *albicans* cells were treated with 4 μg of CaGA at 37 °C for 60 min. Untreated samples were processed in parallel as controls by resuspending the pellet in PBS at the same cell density. Both treated and control samples were immobilized onto sterile glass coverslips precoated with 0.1% aqueous poly-L-lysine (Sigma, USA) to facilitate cell adherence. Prepared samples were then visualized and imaged using a Zeiss EVO 60 Scanning Electron Microscope equipped with an Oxford EDS detector (Zeiss, USA).

#### Nile Red staining

To assess the membrane-associated hydrophobic domains and intracellular lipid content upon CaGA treatment, Nile Red (NR) staining was performed as described previously, with slight modifications.^21^ *C. albicans* cells were cultured in SDB at 30 °C until mid-log phase (OD_600_ ≈ 0.6–0.8). Cells were harvested by centrifugation at 5000 rpm for 5 min, washed twice with PBS, and resuspended to a final density of ∼1 × 10^8^ CFU/mL. Cell suspensions were then incubated with NR (Sigma-Aldrich, USA) at a final concentration of 1 μg/mL in the dark at room temperature for 10 min. After staining, cells were washed twice with PBS to remove excess dye. Fluorescence imaging was performed using a fluorescence microscope equipped with a TRITC or rhodamine filter set (Zeiss Microsystems, USA). The resultant differential fluorescence intensity between treated and control groups was confirmed as indicative of lipid perturbation or ergosterol interaction.

#### Isothermal titration calorimetry

An Isothermal Titration Calorimetry (ITC) experiment was performed using the ITC200 system (GE Healthcare, USA) equipped with non-reactive Hastelloy sample cells to ensure chemical resistance. The sample cell had a volume of 200 μL, while the syringe held a maximum of 39 μL. All titrations were carried out at 298 K (25 °C) using PBS buffer. The experiment involved the titration of a 0.1 mM solution of purified CaGA into a 0.2 mM solution of ergosterol. Before titration, all solutions were thoroughly degassed to eliminate air bubbles and to ensure uniform reaction conditions. The titration protocol included 180-s intervals between injections and a stirring speed of 300 rpm to maintain consistent mixing throughout the experiment. Blank titrations (buffer into 0.04% DMSO) were also performed under identical conditions to counteract the effects of dilution from heat. The binding constant (*K*) and enthalpy change (*ΔH*) were derived following data analysis as described earlier.^22^

#### System setup and steered molecular dynamics simulations

To construct a biologically relevant model of the *C*. *albicans* membrane, a bilayer system was assembled consisting of 136 lipids in the outer leaflet and 100 lipids in the inner leaflet, reflecting an asymmetric distribution consistent with *C*. *albicans* plasma membrane composition (Table 1). The bilayer was generated using the Membrane Builder module of the CHARMM-GUI webserver.^23^ The structure of AmB was retrieved from the PubChem database (CID: 5280965), while the topology and force field parameters were derived using CGenFF within CHARMM-GUI.^24^^,^^25^ The simulation box was constructed as a tetragonal unit cell measuring 8.2 × 8.2 × 8.5 nm^3^ along the x-, y-, and z-axes. AmB and modeled CaGA were inserted above the outer leaflet of the bilayer to mimic initial membrane-contact scenarios (Figure S1). Next, the system was solvated using TIP3P water molecules, and ionic strength was adjusted to 0.15 M by adding Na^+^ and Cl^−^ ions to neutralize the system. All MD simulations were performed using GROMACS 2020. Energy minimization was carried out using the steepest descent algorithm for 50,000 steps, followed by two phases of 10 ns NVT equilibration and multiple rounds of 20 ns NPT equilibration. During the equilibration, positional restraints on lipids, ligand heavy atoms, and protein backbones were gradually reduced. Long-range electrostatics were handled using the Particle Mesh Ewald (PME) method. At the same time, Lennard-Jones and short-range electrostatic interactions were computed using the Verlet cutoff scheme with a cutoff radius of 1.2 nm.^26^ Thermodynamic equilibration was maintained using the Berendsen thermostat and barostat, with coupling times of 1 ps (temperature) and 5 ps (pressure) at 303.15 K and 1 bar, respectively.^27^ A 2 fs time step was employed throughout all simulations. Covalent bonds involving hydrogen atoms were constrained using the LINCS algorithm, and water geometry was maintained using the SETTLE algorithm.^26^ For production runs, temperature and pressure coupling were switched to the Nosé–Hoover and the Parrinello-Rahman barostat, respectively, for more accurate sampling of the NPT ensemble.^28^ The equilibrated bilayer systems were subjected to Steered Molecular Dynamics (SMD) simulations. CaGA and AmB were pulled along the bilayer normal (z axis) at a constant velocity of 0.1 Å/ns, simulating their translocation from the outer to the inner membrane interface while a harmonic force constant of 100 kJ mol^−1^ nm^−2^ was applied during pulling. Visualization and rendering of molecular trajectories and membrane interactions were performed using VMD and PyMOL.^29^

**Table 1:** Lipid composition for the asymmetric plasma membrane of *C*. *albicans*

Lipids	Lipid heads/tails	#Lipids in leaflets		Charge
		Outer	Inner	
DYPC	PC(16:1(9Z)/16:1(9Z))	8	10	0
YOPC	PC(16:1(9Z)/18:1(9Z))	3	6	0
POPE	PE(16:0/18:1(9Z))	3	10	0
PYPE	PE(16:0/16:1(9Z))	1	5	0
YOPE	PE(16:1(9Z)/18:1(9Z))	3	6	0
POPI	PI(16:0/18:1(9Z))	5	20	−1
POPS	PS(16:0/18:1(9Z))	7	31	−1
YOPA	PA(16:1(9Z)/18:1(9Z)	3	8	−1
ERG	Ergosterol	49	4	0
MIPC	MIPC(d18:1/16:0)	54	0	0

#### Statistical analysis

All data are presented as mean ± standard deviation (SD) from three independent biological experiments, with each experiment performed in triplicate unless otherwise stated. Statistical analyses were performed using GraphPad Prism 5. Normality of datasets was assessed using the D’Agostino-Pearson omnibus normality test. For parametric data, statistical significance was determined using two-way ANOVA followed by Bonferroni’s multiple-comparison test. For datasets that did not meet normality assumptions, appropriate non-parametric analyses were applied, including one-sample t-tests where applicable. A *p*-value of <0.05 was considered statistically significant. Details of statistical tests, exact *n* values, and significance information are provided in the corresponding figure legends and Results section.
