# Supplementary material for: Cardio-green butyl acetate targets ergosterol to kill Candidaalbicans
Source: iScience. 2026 Jul 24;29(8):116886. doi: 10.1016/j.isci.2026.116886 (PMC13427575; doi:10.1016/j.isci.2026.116886)

iScience, Volume *29*

Supplemental information

Cardio-green butyl acetate targets ergosterol to kill *Candida albicans*

Piyush Baindara, Suresh K. Mondal, Dinata Roy, Sourav Chakraborty, Dheeraj Kumar Sarkar, Santi M. Mandal, and Gourisankar Ghosh

Figure S1. Cardiogreen Complex. ^{1}HNMR: 1.62-1.69, 6H (m), 1.84-1.86, 3H (m), 2.07, 6H (S), 2.08, 4H (S), 2.46-2.56, 8H (m), 2.75, 2H (S), 2.89, 2H (S), 3.29, 16H (S), 3.51-3.54, 4H (t), 4.02, 2H (S), 5.02-5.06, 1H (m), 5.26-5.42, 1H (m), 6.02-6.06, 2H (m), 6.42-6.49, 2H (m), 7.74-7.86, 5H (m), 7.93-8.21,5H (m).

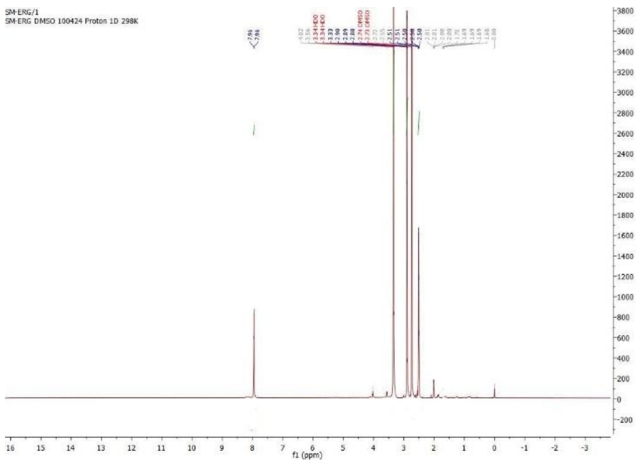

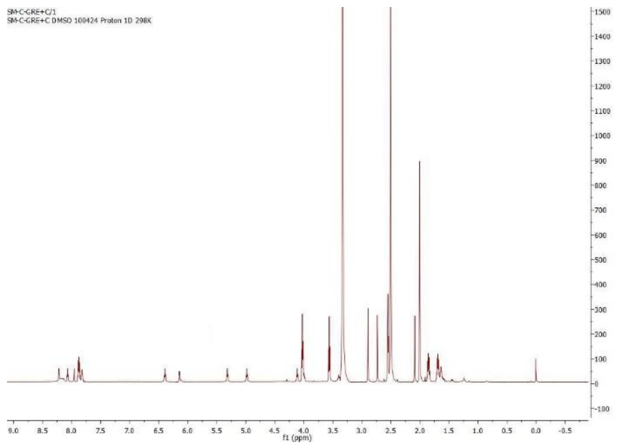

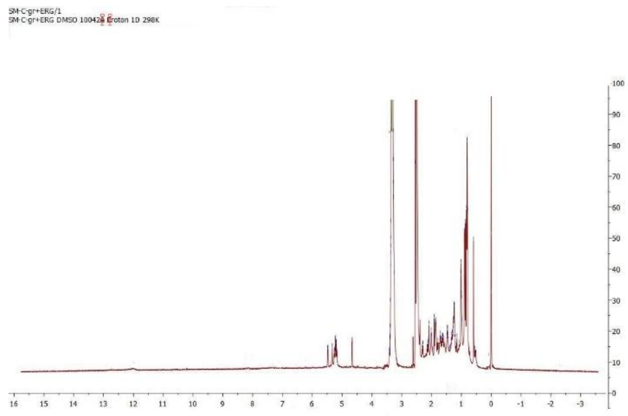

Supplement: Document S1. Figure S1 [file mmc1.pdf]
